# Does Consumption of LC Omega-3 PUFA Enhance Cognitive Performance in Healthy School-Aged Children and throughout Adulthood? Evidence from Clinical Trials

**DOI:** 10.3390/nu6072730

**Published:** 2014-07-22

**Authors:** Welma Stonehouse

**Affiliations:** CSIRO Food and Nutrition Flagship, P.O. Box 10041, Adelaide BC, South Australia 5000, Australia; E-Mail: welma.stonehouse@csiro.au; Tel.: +61-8-8303-8919; Fax: +61-8-8303-8899

**Keywords:** long-chain omega-3 polyunsaturated fatty acids, docosahexaenoic acid, DHA, cognitive performance, healthy, children, adults, older adults

## Abstract

Long-chain (LC) omega-3 PUFA derived from marine sources may play an important role in cognitive performance throughout all life stages. Docosahexaenoic acid (DHA), the dominant omega-3 in the brain, is a major component of neuronal cell membranes and affects various neurological pathways and processess. Despite its critical role in brain function, human’s capacity to synthesize DHA *de novo* is limited and its consumption through the diet is important. However, many individuals do not or rarely consume seafood. The aim of this review is to critically evaluate the current evidence from randomised controlled trials (RCT) in healthy school-aged children, younger and older adults to determine whether consumption of LC omega-3 PUFA improves cognitive performance and to make recommendations for future research. Current evidence suggests that consumption of LC omega-3 PUFA, particularly DHA, may enhance cognitive performance relating to learning, cognitive development, memory and speed of performing cognitive tasks. Those who habitually consume diets low in DHA, children with low literacy ability and malnourished and older adults with age-related cognitive decline and mild cognitive impairment seem to benefit most. However, study design limitations in many RCTs hamper firm conclusions. The measurement of a uniform biomarker, e.g., % DHA in red blood cells, is essential to establish baseline DHA-status, to determine targets for cognitive performance and to facilitate dosage recommendations. It is recommended that future studies be at least 16 weeks in duration, account for potential interaction effects of gender, age and apolipoprotein E genotype, include vegan/vegetarian populations, include measures of speed of cognitive performance and include brain imaging technologies as supportive information on working mechanisms of LC omega-3 PUFA.

## 1. Introduction

Optimal cognitive performance is vital throughout all stages of life. During childhood it is critical to optimize brain development; throughout adulthood it is important to maintain optimal cognitive functioning; and during old-age it is imperative to defer cognitive decline and prevent dementia. The long-chain (LC) omega-3 polyunsaturated fatty acids (PUFA) derived from marine sources, docosaehexaenoic acid (C22:6, DHA) and eicosapentaenoic acid (C20:5, EPA), may play an important role in achieving these objectives. DHA is the dominant LC omega-3 PUFA in the brain [1] and has been shown to accumulate in areas of the brain associated with learning and memory, such as the cerebral cortex and hippocampus [2,3]. DHA is incorporated into neuronal membrane glycerophospholipids at the *sn*-2 position where it regulates numerous neuronal and glial cell processes including neurogenesis, neuroplasticity, neurite outgrowth, synaptogenesis and membrane fluidity which in turn supports membrane protein functions impacting on speed of signal transduction and neurotransmission [4,5,6,7,8]. In addition, DHA improves vascular tone which results in increased cerebral blood flow during cognitive tasks [9] and it regulates the transport and uptake of glucose by the endothelial cells of the blood brain barrier [7,10]. Unesterified DHA released from glycerophospholipids by phospholipase A2 are natural ligands for several nuclear receptors that regulate gene expression, and they are precursors for neuroprotectins and resolvins that counteract neuroinflammation, oxidative stress and increases neuronal survival [4,8,10]. EPA and the plant derived omega-3 fatty acid, alpha-linolenic acid (ALA), also crosses the blood brain barrier, but >99% of these fatty acids are β-oxidised and some EPA is de-esterified from glycerophospholipids [11]. Both ALA and EPA may contribute to brain function by facilitating fuel supply to the brain through ketogenesis, particularly during aging [12]. In addition, unesterified EPA may further contribute to cognitive function through the synthesis of eicosanoids that offset neuroinflammation and improve cerebral blood flow due to its antithrombotic and vasodilatory properties [7]. Thus, DHA seems to be more important for brain function, but ALA and EPA also play minor roles.

The brain retains its DHA content as indicated by a long DHA half-life of ~2.5 years in human brain [13], but studies in animal models have shown that long-term DHA depletion results in significant losses in brain DHA [2,14]. Studies in rats which involved dietary LC omega-3 PUFA depletion over successive generations or even in one-generation showed decreased brain DHA levels, particularly in the frontal cortex and hippocampus areas, with reciprocal increased levels of the omega-6 PUFA, docosapentaenoic acid (DPA, C22:5, *n*-6). With the increase in DPA the level of unsaturation was maintained, but brain function was impaired, including changes in learning, memory, auditory and olfactory responses [2,6,14]. These effects were, however, restored by repletion with dietary DHA [14]. Thus, individuals who follow omega-3 PUFA deficient diets particularly over several generations, for example families who never consume seafood, the major source of DHA, may have depleted levels of brain DHA and their cognitive function may not be optimal. Based on studies in rodents and non-human primates the brain has the capacity to nearly meet its daily needs for DHA through the conversion of ALA to DHA, mainly by the liver, when sufficient dietary ALA (>1.2 g ALA/day) is consumed [15]. The capacity of humans to synthesise DHA *de novo* is limited. While DHA is retro-converted to EPA; the extent of conversion of EPA and ALA to DHA is small [1]. The conversion of ALA to DHA is influenced by several factors; a background diet high in linoleic acid (LA, C18:2, *n*-6) reduces the conversion due to substrate competition; the conversion is more efficient in women [1]; and low dietary intakes of DHA increases conversion [15]. Vegans and vegetarians seem to have similar capacity to convert ALA to DHA than omnivores with ALA supplementation increasing EPA to a small extent, with little effect on DHA in blood lipids [16,17]. Consumption of preformed DHA from fish and seafood, supplements (marine or algae derived) or DHA enriched foods may therefore be a more efficient way of ensuring adequate supply of DHA for optimal brain development and function. Large proportions of populations consume inadequate amounts of LC omega-3 PUFA and fish and seafood. The 2008/2009 New Zealand Adult Nutrition Survey reported that ~30% of adults did not or rarely consumed seafood [18]. Only 21% of Australian children consumed fish or seafood during the two-day Australian National Children’s Nutrition and Physical Activity survey [19] and Australian adults reported average consumption of 0.25 g/day of total LC omega-3 PUFA (including EPA, DHA and docosapentaenoic acid (DPA)) [20]. Median usual intakes of total fish and fish high in LC omega-3 PUFA reported by U.S. adults was 12.2 and 1.98 g/day, respectively and median intakes of DHA from foods plus dietary supplements was only 0.04 g/day [21]. Belgian adults reported median DHA intakes ranging from 0.07 to 0.09 g/day [22]. Several studies have shown that vegan diets are devoid of DHA and vegetarian diets that include dairy food and eggs only provide about 0.02 g DHA/day (reviewed by Sanders [16]). These low intakes were accompanied by substantially lower levels of DHA in plasma, serum, red blood cells (RBC) and plasma phospholipids (PL) in vegans and vegetarians compared to omnivores [16]. Although populations following DHA deficient diets do not seem to exhibit apparent cognitive dysfunction, it is imperative to acertain whether increased consumption of DHA by individuals with low dietary intakes, but otherwise healthy will enhance cognitive performance. In summary, basic research provides strong support for the notion that LC omega-3 PUFA, particularly DHA, play an important role in brain function; but will consumption of LC omega-3 PUFA enhance cognitive performance in healthy school-aged children and throughout adulthood, particularly in populations with low dietary intakes? This review will aim to answer this question by critically examining the evidence from all the clinical trials that have been conducted on healthy school-aged children, younger adults and older adults investigating the effects of LC omega-3 PUFA on cognitive performance. Recommendations for future research will also be made.

## 2. Evidence from Clinical Trials in Healthy Mainstream School-Aged Children

DHA may be particularly important during periods of brain growth spurts which take place from the last trimester of pregnancy up to 2 years of age. Thereafter, the frontal lobes continue to develop throughout childhood, adolescence and into the late twenties with spurts of frontal lobe development at age 7–9 years and mid-adolescence [23,24]. Table 1 provides a summary of all nutrition intervention trials that have investigated the effects of LC omega-3 PUFA on cognitive function, learning and school achievement in healthy school-aged children. Kuratko *et al*. [25] have also reviewed studies published until November 2012 on DHA and learning and behavior in healthy children. The evidence from clinical trials focusing on healthy mainstream school-aged children is relatively new as evident from the small number of studies (*n* = 10) published since 2007. Most of the studies were conducted in children aged 6–12 years old. The studies varied widely with regard to duration (from 8 weeks to 12 months), dosage (from 0.1 to 1.2 g LC omega-3/day), type of LC *n*-3 PUFA (fish oil, algal oil, enriched foods) and DHA:EPA ratio of the interventions, type of outcomes measured and type of participants. LC omega-3 PUFA was generally provided in the form of fish oil or algal oil (high in DHA) and in a few studies as LC omega-3 PUFA enriched foods. Most studies were conducted in children with low baseline intakes of LC omega-3 PUFA. A landmark study by McNamara *et al*. [26] showed for the first time in humans the direct link between DHA and brain activation. Supplementation of 0.4 g/day and 1.2 g/day of DHA increased activation of the dorsolateral prefrontal cortex during a sustained attention task in boys aged 8–10 years. However, these effects were not translated into improvements in visual sustained attention performance. Eight weeks may be sufficient for DHA to increase brain activation, but longer periods may be needed to result in improved cognitive performance.

Richardson *et al*. [27] showed that DHA supplementation improved reading in children who underperformed in reading. Children with reading scores ≤20th centile gained an additional 0.8 months in reading age while children in the ≤10th centile gained 1.9 months in reading age with DHA supplementation. Parletta *et al*. [28] showed in Australian indigenous children with low literacy ability improvements with EPA + DHA on cognitive development (draw-a-person) with a larger effect in the 7–12 year old children. They were unable to show improvements in academic achievement (reading and spelling). However, these results should be interpreted in context of the myriad of factors in this indigenous population that may have affected the attainment of English literacy, e.g., language experiences, home support, socio-economic status (SES) and school attendance. Omega-3 supplementation alone may not have been sufficient to overcome these factors. The fact that improvements were seen in the draw-a-person variable, a non-verbal, culture-free test of cognitive development that does not require schooling or specific language skills, supports this argument [28]. Studies in malnourished 7–9 year old South African [29] and 8–12 year old Mexican children [30] showed improvements in learning and cognitive performance with LC omega-3 supplementation. Whereas no effects were seen over 12 months in 6–10 year old malnourished children from India [31] and Indonesia [32]. However, dosages were small and the supplement used in the Indian study was mostly ALA [31,32]. Baumgartner *et al*. [33] conducted the first LC omega-3 trial in children who were purposely recruited with iron deficiency; they showed that EPA + DHA supplementation in children with iron deficiency anemia had negative effects on working memory. They also showed treatment gender interactions where boys with iron deficiency performed better in long-term memory and retrieval with DHA while girls performed worse. These studies demonstrate the complexities of conducting omega-3 supplementation trials on cognitive performance and learning in malnourished populations where multiple other factors and nutrient deficiencies may affect the outcomes. Yet, these are the populations most likely to benefit from supplementation.

Two studies conducted in healthy mainstream school children in the UK did not show any benefit of consuming DHA on cognitive performance and learning [34,35]. The study by Kennedy *et al*. [34] was underpowered and short in duration (8 weeks). In Kirby *et al*. [35], cheek cell EPA and DHA increased in both DHA and placebo groups, although the increase was greater in the DHA group. Thus, parents of children in the placebo group may have become more aware of the benefits of omega-3 PUFA and increased their intakes.

Inconsistencies between studies could be ascribed to potential modulating effects of age and gender. Children at different phases of brain and cognitive development and boys and girls may respond differently to LC omega-3 PUFA supplementation as was shown by Parletta *et al*. [28] and Baumgartner *et al*. [33]. In a large sample of 6–16 year old American children, the relationship between dietary omega-3 PUFA and cognitive test scores was twice as strong in girls as in boys [36]. None of the other studies reviewed investigated potential interaction effects of age and gender and some studies used wide age ranges which may have resulted in greater variability or response modulating effects on outcomes.

Biomarkers of LC omega-3 PUFA intake are often not measured in studies on children because of children’s fear of having a blood sample taken and consequently not wanting to volunteer for studies. Cheek cell samples are sometimes collected, which is much less invasive and has been shown to correlate well with dietary intakes, plasma and RBC levels [37]. The studies in Table 1 measured DHA and EPA levels/concentrations in RBC, plasma, RBC or plasma phospholipids (PL) and cheek cells. The levels increased in all studies with supplementation of LC omega-3 PUFA and the magnitude of the increase often reflected the supplementation dose [26,29,31,32,33,35].

To date, no LC omega-3 PUFA interventions have been conducted in adolescents and the only evidence is from observational studies. A prospective study in >9000 15 year old Swedish school children showed significantly higher school grades at age 16 in adolescents consuming fish more than once a week compared to less than once a week [38]. At age 18, male adolescents who consumed fish more than once per week compared to less than once per week at age 15 had higher IQ scores which was obtained from completed intelligence tests as part of the mandatory Swedish military service conscription examination [39]. De Groot *et al*. [40] recently showed in 700 Dutch adolescents, 12–18 years, that consumption of fish providing the recommended amount of EPA + DHA of ~0.45 g/day compared to no fish intake was associated with more advanced vocabulary and higher end term grades. However, eating more fish than the recommended amount was no more beneficial [40].

In summary, it seems as if children with low literacy ability and who are malnourished with low LC omega-3 PUFA intakes may benefit most from the consumption of LC omega-3 PUFA with regard to cognitive outcomes (e.g., memory, non-verbal cognitive development, processing speed, visual-perceptive capacity, attention and executive function) and school achievement (e.g., reading and spelling). Inconsistencies between studies may have been due to different dosages, duration, other nutrient deficiencies and lack of investigating interaction effects of gender and age. Dosages may have been too low in several of the studies that did not show benefits. Nutrient deficiencies such as iron deficiency in malnourished populations may need to be corrected before supplementation with LC omega-3 fatty acids can commence to avoid potential adverse interaction between nutrient deficiencies and omega-3 supplementation.

**Table 1:** Nutrition intervention trials of long-chain omega-3 PUFA and cognitive function, learning and school achievement in healthy school-aged children.

Reference	Study Design	Participants	Intervention	Results	
				Cognitive Performance (LC Omega-3 *vs*. Placebo)	Biomarker
Baumgartner *et al*. 2012 [33] ^a^	RCT, 8.5 months KwaZulu-Natal, South Africa	6–11 years, low-income iron deficient children (*n* = 321, analysed *n* = 288). Excluded children consuming omega-3 supplements. Low baseline omega-3 status based on low RBC DHA (~3%) and EPA (~0.16%)	Four interventions as supplements: (1) Iron + fish oil (2) Iron + placebo; (3) Fish oil + placebo; (4) Placebo + placebo. Fish oil = 0.5 g/day LC omega-3 (0.42 g DHA + 0.08 g EPA). Provided 4 days/week at school.	No effects on cognitive outcomes. LC omega-3 PUFA without iron had negative effects on working memory in children with iron deficiency anaemia and on long-term memory and retrieval in girls with iron deficiency, whereas boys with iron deficiency performed better.	RBC DHA increased by 2.21% (to ~5.9%); RBC EPA increased by 0.14% (to ~0.38%).
Dalton *et al*. 2009 [29]	RCT, 6 months Northern Cape, South Africa	7–9 years, low-income, marginally nourished indigenous children (*n* = 183, analysis on *n* = 155)	Fish flour bread spread provided at school (~0.89 g/week DHA (0.13 g/day)) *vs*. control bread spread.	Improved verbal learning ability and memory. Tendency to improve reading (*p* = 0.06). Prevented decline in spelling. Secondary analysis: effects more pronounced in children with lower baseline performance scores.	Increased EPA and DHA in plasma PC, RBC PC, RBC PE.
Kennedy *et al*. 2009 [34]	RCT, 8 weeks Newcastle-upon-Tyne area, UK	10–12 years (*n* = 90, analysis on *n* = 86). Excluded children with high intake of LC omega-3 sources	Three intervention arms: (1) Low dose algal oil: 0.4 g DHA (2) High dose algal oil: 1.0 g/day DHA (3) Placebo (vegetable oil)	No effects on comprehensive computerized cognitive test battery (including memory, working memory, attention, and reaction time) Word recognition task: Low dose: faster performance; High dose: slower performance	NR
Kirby *et al*. 2010 [35]	RCT, 16 weeks Newport, UK	8–10 years (*n* = 450, analysis on *n* = 348). Excluded children consuming omega-3 supplements.	Fish oil: (0.4 g DHA + 0.06 g EPA)/day + micronutrients *vs*. placebo (olive oil)	No effects on comprehensive cognitive performance test battery: (IQ, reading & spelling, working memory, attention, impulsivity, handwriting)	Cheek cell fatty acids: EPA and DHA increased in both DHA and placebo groups with greater increase in DHA group.
McNamara *et al*. 2010 [26]	RCT, 8 weeks Cincinnati, OH, USA	8–10 year boys (*n* = 38, analysis on *n* = 33). Low baseline omega-3 status based on low RBC DHA (3.3%).	Three intervention arms: (1) Low dose algal oil: 0.4 g/day DHA (2) High dose algal oil: 1.2 g/day DHA (3) Placebo (corn oil)	Both dosages increased activation of the dorsolateral prefrontal cortex during sustained attention task. No effect on attention or reaction time of attention.	RBC DHA increased by ~4.2% (to 7.5%) in low dose and by ~7% (to 10.3%) in high dose
Muthayya *et al*. 2009 [31]^a^	RCT, 12 months Bangalore, India	6–10 years, low income, marginally nourished (*n* = 598, analysis on *n* = 550) Low baseline omega-3 status based on low RBC DHA (3.2%) and EPA (0.18%)	Four interventions provided as fortified foods: (1) High micronutrients + high omega-3 (2) High micronutrients + low omega-3 (3) Low micronutrients + high omega-3 (4) Low micronutrients + low omega-3 Low dose: 0.14 g/day ALA High dose: (0.93 g ALA + 0.10 g DHA)/day	No effects on mental processing, memory, fluid reasoning, retrieval ability or cognitive speediness.	High *vs*. low dose difference: RBC DHA increased by 1.55% (to ~5.2%); RBC EPA increased by 0.11% (to ~0.37%)
Osendarp *et al*. 2007 (NEMO Study Group) [32] ^a^	RCT, 12 months Adelaide, Australia and Jakarta, Indonesia	6–10 years. Australia: well nourished, (*n* = 396, analysed *n* = 276) Indonesia: marginally nourished, (*n* = 384, analysed *n* = 367) Baseline plasma omega-3: Australia: EPA ~7.6 μg/mL, DHA ~33 μg/mL Indonesia: EPA ~1.2 μg/mL, DHA ~41 μg/mL	Four interventions provided as flavored drinks: (1) High micronutrients (2) DHA + EPA (0.09 g DHA + 0.02 g EPA)/day (3) Micronutrients + DHA + EPA (as above) (4) Placebo	No effects on general intelligence, verbal learning and memory or visual attention	Australia: Increased plasma DHA with 11.1 μg/mL, EPA 2.23 μg/mL. Indonesia: Increased plasma DHA with 7.06 μg/mL
Parletta *et al*. 2013 [28]	RCT (20 weeks) with one-way cross-over to LC omega-3 supplement (20 weeks) Northern Territory, Australia	3–13 years, indigenous children with low literacy ability (*n* = 408). Low intakes of omega-3 rich fish	Fish oil: 0.75 g LC omega-3 per school day (0.56 g EPA + 0.17 g DHA) plus 0.06 g/day gamma linolenic acid *vs*. placebo (palm oil)	Reading & Spelling: No effect. Non-verbal cognitive development (Draw-A Person): Improvements with strongest effects in 7–12 year olds	NR
Portillo-Reyes *et al*. 2014 [30]	RCT, 3 months low SES schools in Ciudad Juarez, Mexico	8–12 years, mild-moderately malnourished (*n* = 59, analysis on *n* = 50). Excluded children consuming omega-3 supplements. Intake of fish low	Fish oil: 0.45 g/day LC omega-3 (0.18 g EPA + 0.27 g DHA) *vs*. placebo (soybean oil)	Improved processing speed, visual-perceptive capacity, attention, executive function (large effect size improvements (Cohen *d* > 0.8) in >50% of children in 11/18 tests) Memory: No effect	NR
Richardson *et al*. 2012 [27]	RCT, 16 weeks Oxfordshire, UK	7–9 years, underperforming in reading (≤33rd centile) (*n* = 362). Excluded children with high intake of LC omega-3 sources	Algal oil: 0.6 g/day DHA *vs*. placebo (corn/soybean oil)	Reading: Baseline reading scores ≤33rd centile: No effect; ≤20th centile (*n* = 224): improved reading; ≤10th centile (*n* = 105): improved reading Working memory: No effect	NR

Abbreviations: ALA, alpha-linolenic acid; DHA, docosahexaenoic acid; EPA, eicosapentaenoic acid; SES, socio-economic status; LC, long-chain; NR, not reported; PC, phosphatidyl choline; PE, phosphatidylethanolamine; RBC, red blood cell; RCT, randomized controlled trial; ^a^ Multiple supplement arms, only omega-3 study arm reported.

## 3. Evidence from Clinical Trials in Healthy Younger Adults

The aim during younger adulthood is to maintain optimal brain function. Although brain development is established, neuroplasticity is ongoing [6]. Only seven studies to date investigated the effects of LC omega-3 PUFA on cognitive performance in younger healthy adults (Table 2). The study by Stonehouse *et al*. [41] in healthy young adults who habitually consumed diets low in DHA, has been one of the largest and longest trials to date and showed that DHA supplementation improved memory and reaction time of memory [41]. This was also the only study so far in healthy young adults that investigated whether gender and apolipoprotein E genotype (*APOE*) modulated the response to LC omega-3 PUFA supplementation. They showed that memory domains were affected differently by DHA in men and women; in women episodic memory improved whereas in men, reaction time of working memory improved compared to placebo [41]. This may be explained by men and women using different problem-solving strategies as indicated by differences in the functional organization of the brain when performing memory tasks [42,43]. *APOE* did not affect responses in the group as a whole but when stratified for gender, improvements in reaction time for working memory and attention with DHA compared to placebo were more pronounced in male *APOE4* allele carriers than in non-carriers. However, this effects needs to be further explored since the study was not statistically powered to investigate the three-way interaction of treatment*gender*APOE [41]. Apolipoprotein E is the primary lipid transporter in brain tissue with carriers of the *APOE4* allelic variant at several fold increased risk of Alzheimer’s disease (~three- and ~15-fold increase in risk in *APOE3/E4* and *APOE4/E4*, respectively, relative to the wild-type genotype) [44,45]. Structural and functional neurological changes are seen in *APOE4* carrier’s decades before the appearance of any cognitive or clinical symptoms [46,47,48]. Surprisingly, young (20–35 years) *APOE4* carriers have been shown to perform better on cognitive tasks than non-carriers have [49,50]. This may be due to compensatory mechanisms being employed by carriers of the *APOE4* allele as suggested by increased brain activation in the frontal and temporal regions of *APOE4* carriers during memory tasks compared to non-carriers [47]. The *APOE4* carriers may compensate by taking longer to complete the cognitive tasks more accurately. Any effect of DHA supplementation in *APOE4* carriers is therefore more likely to be seen in tasks assessing reaction time as was seen in our study [41]. Considering the relatively high prevalence of *APOE4* carriers, (~24% in Caucasian populations [44,45] and 31% in the New Zealand sample [41]), it may be an important factor to take into account when investigating the cognitive benefits of LC omega-3 PUFA.

None of the other RCTs summarized in Table 2 showed any cognitive benefits with LC omega-3 PUFA [51,52,53,54,55]. Fontani *et al*. [56] showed improvements in sustained attention and reaction time of sustained attention. However, although the trial is described as a RCT, the authors do not report the placebo results and these results should thus be interpreted with caution. None of these trials examined gender or *APOE* interactions. If gender or *APOE* dimorphisms exist, combining groups may cancel out or dilute any potential effects. Some studies used smaller DHA dosages [51,52], had small sample sizes [51,53,54,56], included a wide age range (18–70 years) [55] and all studies were short in duration ranging from 4 to 12 weeks [51,52,53,54,55,56].

**Table 2:** Nutrition intervention trials of long-chain omega-3 PUFA and cognitive function in healthy younger adults.

Reference	Study Design	Participants	Intervention	Results	
				Cognitive Performance (LC Omega-3 *vs*. Placebo)	Biomarker
Antypa *et al*. 2009 [51]	RCT, 4 weeks Leiden, The Netherlands	University students, mean age ~22 years (*n* = 56, analysed *n* = 54) Excluded adults consuming fish more than once/week Baseline plasma DHA: ~1.8%, EPA: 0.48%	Fish oil: 2.3 g/day LC omega-3 (1.74 g EPA + 0.25 g DHA) *vs*. placebo (olive oil)	No effects on attention, memory or reaction time of attention	Increased plasma DHA by ~0.67% (to 2.6%) and EPA by ~2.3% (to 2.84%)
Fontani *et al*. 2005 [56]	RCT (But placebo results not reported), 35 days Siena, Italy	22–51 years (*n* = 33). LC omega-3 intake not considered	Fish oil: 2.8 g/day LC omega-3 (1.6 g EPA + 0.8 g DHA)	Improvements in sustained attention and reaction time of sustained attention	Poorly reported
Jackson *et al*. 2012 [52]	RCT, 12 weeks Newcastle upon Tyne, UK	18–35 years (*n* = 159, analysed *n* = 140) Low average intake of fatty fish (<2 portions/month) Baseline serum DHA: ~1.1%, EPA: ~1.1%	Three intervention arms: (1) DHA-rich fish oil: (0.45 g DHA + 0.09 g EPA)/day (2) EPA-rich fish oil: (0.2 g DHA + 0.3 g EPA)/day (3) Placebo (olive oil)	No effects on comprehensive computerized cognitive test battery (episodic memory, working memory, attention, reaction time, executive function)	DHA group: Increased serum DHA by ~0.61% (to 1.87%) and EPA by ~ 0.31% (to 1.36%) EPA group: Increased DHA by ~0.37% (to 1.49%) and EPA by ~0.62% (to 1.78%)
Jackson *et al*. 2012 [53]	RCT, 12 weeks Newcastle upon Tyne, UK Primary outcome was cerebral blood flow	18–29 years (*n* = 65) Excluded consumers of oily fish and omega-3 supplements	Three intervention arms: (1) Low dose DHA fish oil: (0.45 g DHA + 0.09 g EPA)/day (2) High dose DHA fish oil: (0.9 g DHA + 0.18 g EPA)/day (3) placebo (olive oil)	Increased cerebral blood flow Cognitive tasks only assessed at end of study using comprehensive computerized cognitive test battery (episodic memory, working memory, attention, reaction time, executive function). Both dosages improved reaction times on two attention tasks, but effects were lost when correcting for multiple testing	NR
Karr *et al*. 2012 [54]	Placebo controlled trial, not randomized, 4 weeks Canada	College students (mean age ~20 ± 2 years) (*n* = 43, analysed *n* = 41) Regular consumers of fish excluded	Fish oil: (0.72 g EPA + 0.48 g DHA)/day *vs*. placebo (coconut oil)	No effects on verbal learning and memory, inhibition and executive control	NR
Rogers *et al*. 2008 [55]	RCT, 12 weeks Bristol, UK Primary outcome was depressed mood	Mildly depressed adults, 18–70 years (average ± SD age 38 ± 14 years) (*n* = 218, analysed *n =* 190) Excluded consumers of >1.5 portions oily fish per week	Fish oil: 1.5 g/day LC omega-3 (0.85 g DHA + 0.63 g EPA) *vs*. placebo (olive oil)	No effects on computerised cognitive test battery (processing speed, reasoning, impulsivity, working memory)	Increased plasma EPA + DHA Mean difference between groups at 12 weeks: 3.16% (2.74%, 3.58%)
Stonehouse *et al*. 2013 [41]	RCT, 6 months Auckland, New Zealand	18–45 years (*n* = 228, analysed *n* = 176) Excluded consumers of >~0.2 g EPA + DHA/week and omega-3 supplements Baseline RBC DHA: ~5%, EPA: ~0.6%	Fish oil: (1.2 g DHA + 0.17 g EPA)/day *vs*. placebo (high oleic acid sunflower oil)	Comprehensive computerized cognitive test battery: Reaction times of episodic memory and working memory improved Gender*treatment interactions: Episodic memory improved in women and reaction time for working memory improved in men Gender*treatment*APOE interactions: greater improvements in reaction time for working memory and attention in men. No effects on accuracy of working memory or processing speed	RBC DHA increased by 2.6% (to ~7.9%); RBC EPA increased by 0.2% (to ~0.81%)

Abbreviations: DHA, docosahexaenoic acid; EPA, eicosapentaenoic acid; *APOE*, apolipoprotein E genotype; LC, long-chain; NR, not reported; RBC, red blood cell; RCT, randomized controlled trial; Narendran *et al*. [57] not included in table because the focus was mechanistic and did not use a RCT study design.

Jackson *et al*. [9,53] investigated the effects of short-term (12 weeks) supplementation of LC omega-3 PUFA on neural tissue activation and cerebral blood flow using near-infrared spectroscopy to assess oxy-hemoglobin and deoxy-hemoglobin in the frontal cortex of adults during performance of cognitive tasks. DHA at low and high dosages [9,53], but not EPA [9], significantly increased oxyhemoglobin and total hemoglobin during several cognitive tasks indicating increased cerebral blood flow.

In summary, the trial by Stonehouse *et al*. [41] overcame the study design limitations mentioned above, namely the intervention period was adequate (6 months); a relatively large DHA dosage was used (1.2 g DHA/day) which resulted in achieving RBC DHA levels of ~8%; sufficient statistical power and gender and *APOE* interactions were investigated. They showed that DHA improved memory and reaction time of memory, demonstrating that younger adults may benefit from consumption of DHA [41].

## 4. Evidence from Clinical Trials in Healthy Older Adults

The main aim for cognitive function during older age is to defer cognitive decline and to prevent dementia. Age-related cognitive decline (ARCD) is decline in cognitive functioning as a consequence of the aging process that is within normal age limits [58]. Mild cognitive impairment (MCI) represents a transitional state between ARCD and dementia, but individuals with MCI are able to function normally in everyday life [59,60]. Clinical trials investigating the effects of LC omega-3 PUFA on cognitive performance in healthy older adults (without dementia) (Table 3) have been inconsistent, with some showing no effects [61,62,63,64] and others showing improvements in different measures of cognitive function, mostly memory [58,59,60,65,66,67,68] as well as executive function [68] and visuospatial learning [58]. The outcomes have been affected by various study design limitations such as high baseline LC omega-3 status, wide variations in cognitive impairment scores with MMSE ranging from 21 to 30, small dosages, short trial duration, insensitive outcome measures, insufficient statistical power, wide age ranges, and lack of investigating potential response modulating effects of age, gender and *APOE*. LC omega-3 PUFA were provided in the form of ethyl esters, algal oil, fish oil, enriched margarine (one study [62]) and krill oil (one study investigating effects on brain activation, not cognitive function [69]).

One of the most rigorously designed trials was unable to show any benefit of LC omega-3 PUFA on a range of cognitive outcomes [61]. The authors argued that the population may have already consumed sufficient LC omega-3 PUFA as evident from relatively high serum DHA:DPA (omega-6) ratios in both treatment groups at 24 months. Unfortunately, the authors did not assess the LC omega-3 status at baseline to confirm this. High intakes of dietary LC omega-3 PUFA of ~0.3 g/day may have also precluded any cognitive benefits with fish oil in the study by Van de Rest *et al*. [64]. In addition, wide ranges of mini-mental scale examination (MMSE) scores from 23 to 30 may have resulted in greater variability in cognitive responses that could have resulted in a null effect. The Alpha Omega trial, which had 2911 patients with stable myocardial infarction, has been the largest and longest (40 months) trial so far conducted. Neither ALA, DHA + EPA, nor a combination of ALA + DHA + EPA affected MMSE scores [62]. The study was designed for CVD as primary outcome and MMSE was used as a secondary measure of global cognitive function. MMSE may not be sensitive for detecting small changes in cognitive function with nutrition interventions in a normal aging population. Furthermore, the effects were investigated against a background where >85% of participants were using lipid lowering and anti-hypertensive drugs which in turn may have affected cognitive function through their effects on cardio-metabolic markers, masking the effects of omega-3 PUFA. The most likely explanation for the lack of cognitive benefits in the study by Stough *et al*. [63] was the low dosage (0.25 g/day DHA from tuna oil) consumed over a short duration of 90 days and a wide age-range of 45–80 years which may have increased the variability in the outcome measures.

Yurko-Mauro *et al*. [58] showed significant improvements in several measures of memory as well as visuospatial learning in older adults with subjective memory complaints and ARCD. The improvement in the paired associate learning (PAL) test was related to a gain of 7 years in age compared to reference data. Their study was sufficient in duration (6 months), provided a large dosage (0.9 g/day DHA), had sufficient statistical power and was conducted in individuals with low habitual intakes of DHA.

Two studies were conducted in older adults with MCI [59,60]. Lee *et al*. [59] showed highly significant improvements in memory in older women with MCI. Their sample size was small (*n* = 35), but the effect size was large with a mean Z-score difference between fish oil and placebo of 0.8 (0.34, 1.26). The differences between this and other studies may be that participants with MCI were recruited, leaving more room for improvement in cognitive test scores, participants were from low socioeconomic background likely to consume low amounts of omega-3 rich fish, and a large dosage (1.3 g/day DHA + 0.45 g/day EPA) was consumed over a long duration (12 months). Furthermore, the study was conducted in women only which may have resulted in a more homogeneous sample. The results are consistent with that of Stonehouse *et al*. [41] who showed improvements in memory in younger women [41]. Sinn *et al*. [60], in their study on older adults with MCI, showed improvements in verbal fluency with a high DHA supplement but not with a high EPA supplement. However, this was the only significant effect out of 11 cognitive assessments and could be due to type 1 error. The lack of effects may have been due to insufficient statistical power. However, the recruitment of large numbers of participants with MCI is not an easy task.

Inconsistencies between studies could be ascribed to response modulating effects of gender, age and *APOE*, but very few studies have investigated these effects. Van de Rest *et al*. [64] identified treatment*APOE interactions and treatment*gender interactions with *APOE4* carriers and men showing improvements in attention compared to placebo.

Improvements have been shown in cognitive performance in older adults over short duration with high dosages [66] which is most likely due to the vascular and antithrombotic effects of EPA and DHA rather than their effects on neurological changes. Nilsson *et al*. [66] showed improvements in working memory with a high dosage of 1.5 g/day EPA + 1.05 g/day DHA over a very short period of 5 weeks. They also showed significant improvements in cardio-metabolic risk markers that were inversely related to performance in working memory. Witte *et al*. [68] also showed an inverse relationship between improvements in executive function and fasting insulin.

**Table 3:** Nutrition intervention trials of long-chain omega-3 PUFA and cognitive function in older adults.

Reference	Study Design	Participants	Intervention	Results	
				Cognitive Performance (LC Omega-3 *vs*. Placebo)	Biomarker
Dangour *et al*. 2010 [61] (OPAL Study)	RCT, 24 months England and Wales	70–75 years, cognitively healthy, MMSE ≥ 24 (median = 29) (*n* = 867, analysis on *n* =748) Excluded adults consuming fish oil	Ethyl ester fish oil: (0.2 g EPA + 0.5 g DHA)/day *vs*. placebo (olive oil)	No effect on global cognitive function, memory, processing speed, executive function, global delay score	Serum fatty acid levels in sub-sample (*n* = 235) at 24 months: EPA and DHA higher *vs*. placebo (EPA: 50 *vs*. 39 mg/L; DHA: 96 *vs*. 71 mg/L)
Geleijnse *et al*. 2012 [62] (Alpha Omega Trial)	RCT, 40 months Netherlands Primary outcome was CVD morbidity and mortality	60–80 years, stable MI patients, MMSE >21 (average ± SD 28 ± 1.6 points) Baseline EPA + DHA intake was low (median intake = ~118 (55–200) mg/day) (*n* = 2911)	Four interventions provided in 20 g/day margarine: (1) 0.4 g/day EPA + DHA (2) 2 g/day ALA (3) EPA + DHA + ALA (4) Placebo	No effect on global cognitive decline as measured with MMSE	Increase in plasma CE EPA and DHA in sub-sample (*n* = 600)
Johnson *et al*. 2008 [65]	RCT, 4 months Boston, MA, USA Primary outcome was eye health	60–80 years, healthy women (*n* = 57, analysed *n* = 49) Dietary intake of DHA ~136 mg/day	Four interventions provided as supplements taken with nutritional energy drink: (1) 0.8 g/day DHA (algal oil) (2) 12 mg/day lutein (3) DHA + lutein (4) Placebo	Verbal fluency (semantic/long-term memory) improved in DHA, lutein and DHA + lutein groups; DHA + lutein improved rate of learning (number of trials to learn a list) and memory in 1 of 6 recall tests (some test close to ceiling); No effects on working memory, processing speed or inhibition	Increase in serum DHA
Lee *et al*. 2013 [59]	RCT, 12 months Cheras, Kuala Lumpur, Malaysia	≥60 years, MCI, MMSE = 26.4 (25–28), middle to low-socioeconomic status (*n* = 36, analysed *n* = 35) Excluded participants consuming omega-3 supplements Baseline plasma EPA ~0.48%; DHA ~4.1%	Fish oil: (1.3 g DHA + 0.45 g EPA)/day *vs*. placebo (corn oil)	Improved memory (short-term memory, working memory, immediate visual memory, delayed recall). No effects on executive function/attention, psychomotor speed, visuospatial skills	Increase in plasma DHA and EPA
Nilsson *et al*. 2012 [66]	RCT, cross-over, 5 weeks, 5 weeks washout Lund, Sweden Aim: Relationship between cognitive and cardiometabolic outcomes	51–72 years, healthy (*n* = 44, analysed *n* = 38 No exclusion based on omega-3 intake Ordinary Swedish diet including meat and fish every week	Fish oil: (1.05 g DHA + 1.50 g EPA)/day *vs*. non-oil placebo in tablet form (dicalcium phosphate, microcrystalline cellulose, magnesium salts of fatty acids)	Treatment–consumption sequence interaction; only first period reported: Improved working memory TG and SBP improved TG, SBP, fasting glucose, TNF-α inversely related to working memory performance	NR
Sinn *et al*. 2012 [60]	RCT, 6 months Adelaide and Brisbane, Australia	>65 years, MCI, MMSE ≥ 22 (average ~27 ± 2.5) (*n* = 50) Excluded participants consuming fish >1/week and omega-3 supplements Baseline RBC EPA ~0.96%, DHA ~4.6%	Three intervention arms: (1) EPA-rich fish oil : (1.67 g EPA + 0.16 g DHA)/day) (2) DHA-rich fish oil: (1.55 g DHA + 0.40 g EPA)/day (3) Placebo (safflower oil)	DHA improved verbal fluency (test of fluid thinking/semantic memory). Only one out of 11 cognitive assessments affected	EPA group: RBC DHA increased by ~0.78% (to 5.34%); EPA by ~3.1% (to 4.06%) DHA group: RBC DHA increased by ~4.1% (to 8.65%); EPA by ~0.86% (to 1.83%)
Stough *et al*. 2012 [63]	RCT, 90 days Melbourne, Australia	45–77 years (average ~56 ± 8.7 years), healthy (*n* = 112, analysed *n* = 75) No exclusion based on intake of LC omega-3 sources Baseline plasma PL DHA: ~3.44%	Tuna oil: (0.25 g DHA + 0.06 g EPA)/day *vs*. placebo (soybean oil)	No effects on comprehensive computerized cognitive test battery (attention, secondary memory, working memory, speed of attention, speed of memory)	Plasma PL DHA increased by ~1.79% (to 5.22%)
Van de Rest *et al*. 2008 [64]	RCT, 26 weeks Wageningen, The Netherlands	≥65 years, cognitively healthy, median (25, 75 percentile) MMSE = 28 [27,28,29], ranged from 23 to 30, (*n* = 302) Excluded participants consuming high LC omega-3 sources Dietary EPA+DHA: ~0.3 g/day Baseline plasma CE EPA + DHA: 1.9% ± 1.0%	Three intervention arms: (1) Low-dose fish oil: (0.26 g EPA + 0.18 g DHA)/day (2) High dose fish oil: (1.09 g EPA + 0.85 g DHA)/day (3) Placebo (oleic acid)	No effects on comprehensive test battery (memory, executive function, attention, sensorimotor speed) Treatment–*APOE4* interactions: Attention improved in *APOE4* allele carriers Treatment–gender interactions: Attention improved in men	Increase in plasma CE EPA + DHA: Low dose: by ~0.95% (to 2.83%); High dose: by ~4.5% (to 6.4%)
Vakhapova *et al*. 2010 [67]	RCT, 15 weeks Tel-Aviv, Israel	50–90 years, non-demented participants with memory complaints, MMSE ≥ 27 (average ~28.5 ± 1.11), (*n* = 157, analysed *n* = 122) No exclusion based on intake of LC omega-3 sources	PS containing LC omega-3: 300 mg PS + 0.08 g (DHA + EPA)/day	Improved verbal immediate recall. No effect on other markers. A subset of participants with higher baseline cognitive status performed better on immediate and delayed verbal recall, learning abilities and time to copy a complex figure	NR
Witte *et al*. 2013 [68]	RCT, 26 weeks Berlin, Germany	50–75 years, MMSE < 26 (average ~29 ± 1.0, ranged from 26 to 30), (*n* = 80, analysed *n* = 65) Fish oil supplement users excluded Most participants consumed fish 1/week. Baseline omega-3 index ~8%	Fish oil: 2.2 g/day LC omega-3 (1.32 g EPA + 0.88 g DHA) *vs*. placebo (sunflower oil)	Improved executive function. No effects on memory, sensorimotor speed and attention. Sub-set who showed greatest increase in omega-3 index showed improved memory. Improved white matter microstructural integrity, grey matter volume in frontal, temporal, parietal and limbic areas. Improvements in executive function associated with peripheral BDNF and inversely with fasting insulin.	Omega-3 index (%RBC DHA + EPA) increased to ~9.6%
Yurko-Mauro *et al*. 2010 [58]	RCT, 24 weeks 19 sites in USA	≥55 years (average ~70 ± 9 years), subjective memory complaints with ARCD, MMSE >26 (*n* = 485) Excluded participants who consumed LC omega-3 supplements or >0.2 g/day DHA Baseline DHA intake: 0.14 g/day	0.9 g/day DHA from algal oil *vs*. placebo (corn + soy oil)	Improved visuospatial learning and episodic memory, immediate and delayed verbal recognition memory. No effect on working memory, executive function, MMSE	Plasma PL DHA increased with 3.2%

Abbreviations: ALA, alpha-linolenic acid; *APOE*, apolipoprotein E genotype; ARCD, age related cognitive decline; BDNF, brain-derived neurotrophic factor; CE, cholesterol esters; DHA, docosahexaenoic acid; EPA, eicosapentaenoic acid; LC, long-chain; MCI: mild cognitive impairment; MMSE, Mini-Mental State Examination; NR, not reported; PS, phosphatidylserine; PL, phospholipid; RBC, red blood cell; RCT, randomized controlled trial; SBP, systolic blood pressure; TG, triglycerides; TNF-α, tumor necrosis factor alpha; ^a^ Multiple supplement arms, only omega-3 study arm reported; Richter *et al*. [70] not included in table because it was not a RCT.

Supplementation with krill oil (0.19 g EPA + 0.09 g DHA/day) and sardine oil (0.49 g EPA + 0.25 g DHA) in 61–72 year old men for 12 weeks resulted in increased activation of the dorsolateral prefrontal cortex during a working memory task using near-infrared spectroscopy and electroencephalography compared to placebo. Krill oil also increased activity during a calculation task in the left frontal area, the dominant area for calculations [69]. Fish oil (1.32 g EPA + 0.88 g DHA/day) supplementation in 50–75 year olds over 26 weeks improved brain white matter microstructural integrity and grey matter volume in frontal, temporal, parietal and limbic brain areas [68].

In summary, many of the RCT had intrinsic design limitations which hamper drawing firm conclusions regarding the efficacy of LC omega-3 PUFA on cognitive performance in healthy older adults. However, the current evidence suggests that DHA may be of benefit for older adults with ARCD and MCI, particularly for improving memory.

## 5. Discussion

Trends are emerging from the current evidence suggesting that consumption of LC omega-3 PUFA, particularly DHA, by healthy school-aged children, and younger and older adults may enhance cognitive performance particularly in those who habitually consume diets low in LC omega-3 PUFA. However, the evidence is inconsistent due to various intrinsic design limitations in many of the RCTs which hamper drawing firm conclusions.

Baseline DHA status may have been an important confounding factor in the available research. Since the human brain tenaciously retains DHA [71], individuals who have been following DHA depleted diets over the long-term are most likely to show cognitive benefits with supplementation whereas individuals with already adequate DHA status may not respond. Although several studies excluded participants based on high intakes of LC omega-3 sources (supplements or seafood), the way that this was assessed and the time periods of intake considered (ranging from 2 weeks to 6 months) differed widely between studies. Some studies included biomarkers of DHA intake to verify low baseline status, but it has mostly been used to confirm compliance to LC omega-3 PUFA interventions. The studies used a wide range of biomarkers and units including RBCs, plasma, plasma PL, serum, plasma cholesterolesters (CE), plasma phosphatidylcholine (PC), RBC PC, RBC phosphatidyletanolamine (PE), cheek cells, expressed as either % of total fatty acids or in concentration units. It is therefore difficult to establish the long-term DHA status of participants and to interpret results across studies.

The use of a uniform biomarker is essential in order to establish baseline DHA status, to determine target levels for optimal cognitive performance as well as threshold levels above which no further benefits are seen. The concept of establishing an omega-3 index for mental health has been suggested by Milte *et al*. [72], based on the omega-3 index for mortality from coronary heart disease developed by Harris and Von Schacky [73]. This index expresses the levels of EPA + DHA in RBC membranes as percentage of total RBC fatty acids and an omega-3 index of ≥8% is associated with the greatest cardio-protection whereas an index of ≤4% is associated with the least protection [73]. Since DHA plays a major role in cognitive performance whereas EPA’s role is probably minor, a DHA-index for cognitive performance could be established. A biomarker reflecting long-term intake of DHA may be more appropriate. Plasma DHA reflects recent intakes whereas plasma PL and RBC DHA reflects long-term DHA intakes [1] but RBC DHA has been shown to be more sensitive to long-term intakes than plasma PL [74]. The biomarker also has to correlate well with brain tissue levels. RBC DHA was shown to be the most efficient biomarker for accumulation of DHA in the baboon neonate brain (RBC DHA, *r* = 0.86; plasma DHA, *r* = 0.58) (reviewed by [74]). However, in studies involving children a less invasive biomarker may be more appropriate such as cheek cell DHA. Cheek cell DHA levels have been shown to correlate well with dietary intakes (*r* = 0.65), plasma (*r* = 0.61) and RBC DHA (*r* = 0.58) levels [37]. In a study on piglets cheek cell DHA correlated well with brain DHA levels (*r* = 0.60), but the correlation was not as good as for plasma (*r* = 0.70) and RBC levels (*r* = 0.72) [75]. The analysis of whole blood collected by finger prick and stored on absorbent paper may also provide a non-invasive, rapid, less costly and reliable method for DHA quantification (correlation between RBC DHA and whole blood spot collected by finger prick, *r* = 0.58) [76]. There may therefore be several potential candidate biomarkers, but RBC DHA may be the preferred biomarker because of the established history of the omega-3 index for coronary heart disease [77]. Equations could to be developed to predict a uniform DHA-index level from these different biomarkers. The uniform measurement of a DHA biomarker/index in RCT could facilitate the establishment of target DHA levels at which cognitive performance is optimal which could then guide dietary intake recommendations. We know from kinetic studies that over a period of 6 months, for every 1 g/day DHA consumed, RBC DHA levels increased by 1% [78]. Arterburn *et al*. [1] showed that plasma PL DHA was highly sensitive to dietary intake of DHA up to doses of ~2 g/day after which the DHA levels approached saturation and increased only incrementally. Identification of factors that predict biomarker responses to DHA consumption would be important to estimate dietary requirements for achieving DHA targets. Flock *et al*. [79] identified increased EPA + DHA dose as the strongest predictor of the omega-3 index (% RBC EPA + DHA); lower baseline omega-3 index levels, older age, lower body weight, increased physical activity with increased dose and female sex predicted greater increases in the omega-3 index. The background diet, particularly the omega-6 PUFA content, may also be an important predictor of RBC DHA response [80] that needs to be investigated.

The duration of studies in this review have also been variable ranging from 4 weeks to 2 years. Studies in animal models showed that recovery of brain DHA levels from a state of depletion is a much slower process compared to other tissues. Rats fed an omega-3 repletion diet containing ALA and DHA after being subjected to a low omega-3 PUFA diet through two generations required 8 weeks to reach DHA levels comparable to rats fed omega-3 PUFA adequate diets whereas DHA was almost completely replete in serum and liver after 2 weeks [81]. In rhesus monkeys that were omega-3 PUFA deficient and fed a DHA rich fish oil diet, DHA in phosphatidylethanolamine of the frontal cortex increased after 2 weeks and stabilized after 12 week [82]. The half-life of DHA in the human brain is ~2.5 years [13]. Umhau *et al*. [13] commented that any potential benefit of increasing brain DHA through dietary change may therefore not be fully manifested in clinical trials of only a few weeks and if such rapid improvements occurred it may rather be due to peripheral actions which indirectly affect brain function [13]. The 5-week study by Nilsson *et al*. [66] is an example of this where improvements in working memory correlated with improvements in cardio-metabolic markers. This may also explain why several short term studies failed to show any effects of cognitive function. Several studies of 16 weeks and longer showed improvements in cognitive performance [27,28,29,30,41,58,59,60,68] which is the minimum time needed for RBC DHA to reach a steady state [1,78]. The brain may not be saturated with DHA after 16 weeks of supplementation, but measurable outcomes may become apparent after 16 weeks.

The outcomes that were improved with LC omega-3 PUFA supplementation in children included verbal learning and memory [29], reading [27,29], spelling [29], non-verbal cognitive development [28] and processing speed, visual-perceptive capacity, attention and executive function [30]; in younger adults memory and reaction time of memory were improved [41]; and in older adults several studies showed improvements in memory [27,28,29,41,58,59,60,65,68], while executive function [68] and visuospatial learning [58] were also improved. Very few studies assessed the speed of performing cognitive tasks. This represents a fundamental measure of brain function and is equally informative or complementary to information on the accuracy of task performance [83]. Speed of information processing is one of the cognitive abilities in children to develop first and is fundamental to the development and expression of other cognitive abilities such as learning, memory and executive functions [23]. Bearing in mind that DHA improves neural communication through several mechanisms as discussed in the introduction, it is highly likely that DHA may affect speed of cognitive performance. Stonehouse *et al*. [41] showed improvements in reaction time of episodic memory and working memory, but not processing speed; Portillo-Reyes *et al*. [30] showed improvements in processing speed; and McNamara *et al*. [26] showed an inverse relationship between RBC DHA levels and reaction time in a sustained attention task while Muthayya *et al*. [31] could not show any effect on cognitive speediness with a ALA supplement containing a small amount of DHA (0.1 g/day). The significance of any speed change should be interpreted in the context of the function that was assessed [84]. Since memory has been the outcome most often shown to be improved by DHA supplementation, it is likely that DHA may also improve the speed at which memory tasks are performed as was shown by Stonehouse *et al*. [41]. It is suggested that future studies include this outcome in their battery of tests. The use of computerized test batteries allows for the assessment of speed of performing cognitive tasks, but also has the advantage of standardized presentation of cognitive tests, it removes the person-to-person interactions with a researcher that can bias and obfuscate data, and it allows for closely controlled collection of a large amount of data within a short period of time [83]. On the other hand, it may be tempting for researchers to assess multiple cognitive outcomes in the hope to find positive results. However, statistical significant findings from this approach are likely to result from chance alone (type 1 error) [85]. Instead, an approach where a small set of cognitive outcomes are identified and pre-specified (primary outcome) [85] based on current evidence, e.g., memory, and investigated in greater detail will be more valuable in substantiating the effects of LC omega-3 PUFA on cognitive performance than a shot-gun approach.

The increased incorporation of brain imaging technologies in future LC omega-3 PUFA interventions could provide valuable supportive *in vivo* information on the working mechanisms of LC omega-3 PUFA. Brain imaging makers can reliably reflect neurostructural, neurophysiological, neurochemical and functional cerebral changes occurring in response to the intervention. However, these imaging markers cannot be considered as a substitute of clinical endpoints in terms of cognitive or behavioral response to a task or challenge [86].

As discussed above, outcomes may have been confounded by potential response modulating effects of gender, age and *APOE*, but very few studies have investigated these interaction effects. If dimorphisms exist for any of these factors, potential effects may be diluted or cancelled out resulting in biased conclusions. Future trials of DHA on cognitive function should take these factors into account by either recruiting homogenous samples or by planning gender-, age- or *APOE*-stratification into the study design to ensure stratified randomization and sufficient statistical power.

To date, no studies have been conducted in vegan and vegetarian populations, who have much lower dietary and blood DHA levels compared to omnivores [16], to determine the association between DHA intake and cognitive function. Sarter *et al*. [17] suggest that lifetime DHA insufficiency may put vegans at increased risk for cognitive dysfunction. It is therefore important that future research studies focus on this target population. The availability of vegetarian omega-3 supplements, e.g., algae-sourced DHA, and evidence that supplementation with these preparation result in increased plasma and RBC DHA levels in vegans and vegetarians [16,17,87] makes this possible.

## 6. Conclusions

Individuals with low habitual intake of LC omega-3 PUFA, children with low literacy ability and who are malnourished, and older adults with ARCD and MCI may benefit most from consuming LC omega-3 PUFA, particularly DHA. However, the evidence-base is still emerging and RCTs have been inconsistent with many study design limitations. A major challenge ahead is the design and conduct of rigorous RCT to provide the evidence-base for dietary recommendations regarding DHA. It is recommended that future studies include a uniform biomarker, e.g., % DHA in RBC, in order to establish baseline DHA-status, determine targets for improved cognitive performance and to facilitate dosage recommendations. It is also recommended that future studies be at least 16 weeks in duration, account for potential interaction effects of gender, age and apolipoprotein E genotype, include vegan/vegetarian populations, include measures of speed of cognitive performance which could be facilitated by using computerised cognitive test batteries and include brain imaging technologies as supportive information on working mechanisms of LC omega-3 PUFA.

Supplementation with DHA is unlikely to be a “magic bullet” that will create geniuses. However, because of humans’ limited capacity to synthesise DHA *de novo* and its critical role in brain function it seems prudent that healthy individuals should include DHA in their diets for optimal cognitive performance through all stages of life. While the evidence is not available yet to make specific recommendations for dietary intake of LC omega-3 PUFA and cognitive performance, we should aim to achieve country specific recommendations of LC omega-3 PUFA. Several international organisations recommend consumption of ≥500 mg/day EPA+DHA or ≥2 fatty fish meals/week [88]. The Australian–New Zealand recommended suggested dietary targets (SDT) for LC omega-3 PUFA is 610 mg/day for men and 430 mg/day for women aged 14 and older [89]. The energy adjusted SDT for 9–13 year old boys and girls are 510 and 410 mg/day and for 4–8 year old boys and girls 400 and 350 mg/day, respectively [90].
